# Supplementary material for: Genomic and Transcriptomic Characterization of Atypical Recurrent Flank Alopecia in the Cesky Fousek
Source: Genes (Basel). 2022 Apr 7;13(4):650. doi: 10.3390/genes13040650 (PMC9033119; doi:10.3390/genes13040650)

Figure S1: Two PCA plots of all individuals collected for the study (n = 216). The PCA plot shows genetic distances between individuals. Figure (a) shows relationships between affected (red) and healthy (blue) individuals and their geographic origin (circular point - individuals from the Czech Republic; triangle point - individuals from the US Club CFNA). Note that individuals kept in the US but born in the Czech Republic were placed in the "CZ" group. Figure (b) shows relationships between three plates of samples genotyped in 2016 (plate 1), 2018 (plate 2), and 2019 (plate 3).

(a)

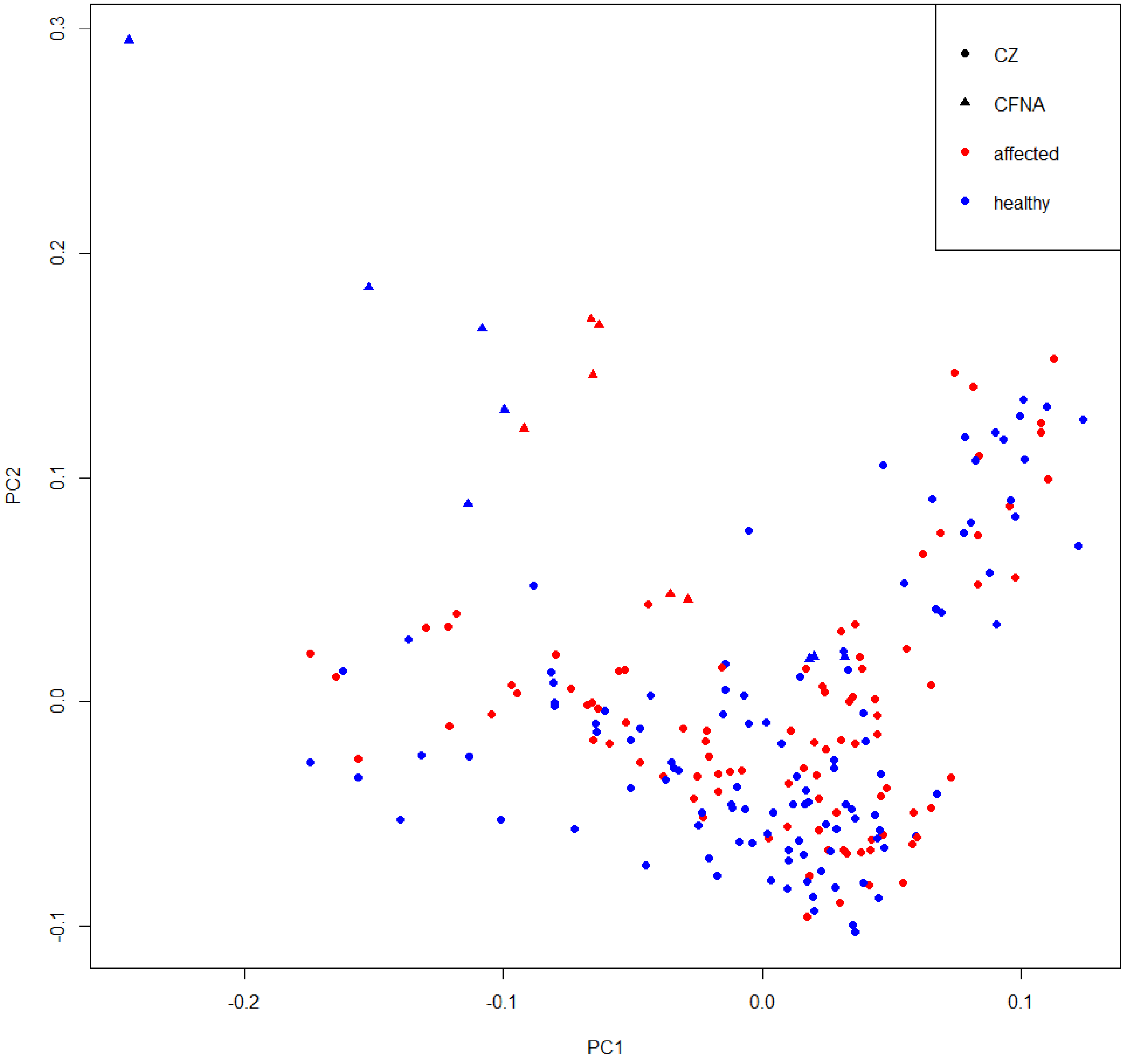

(b)

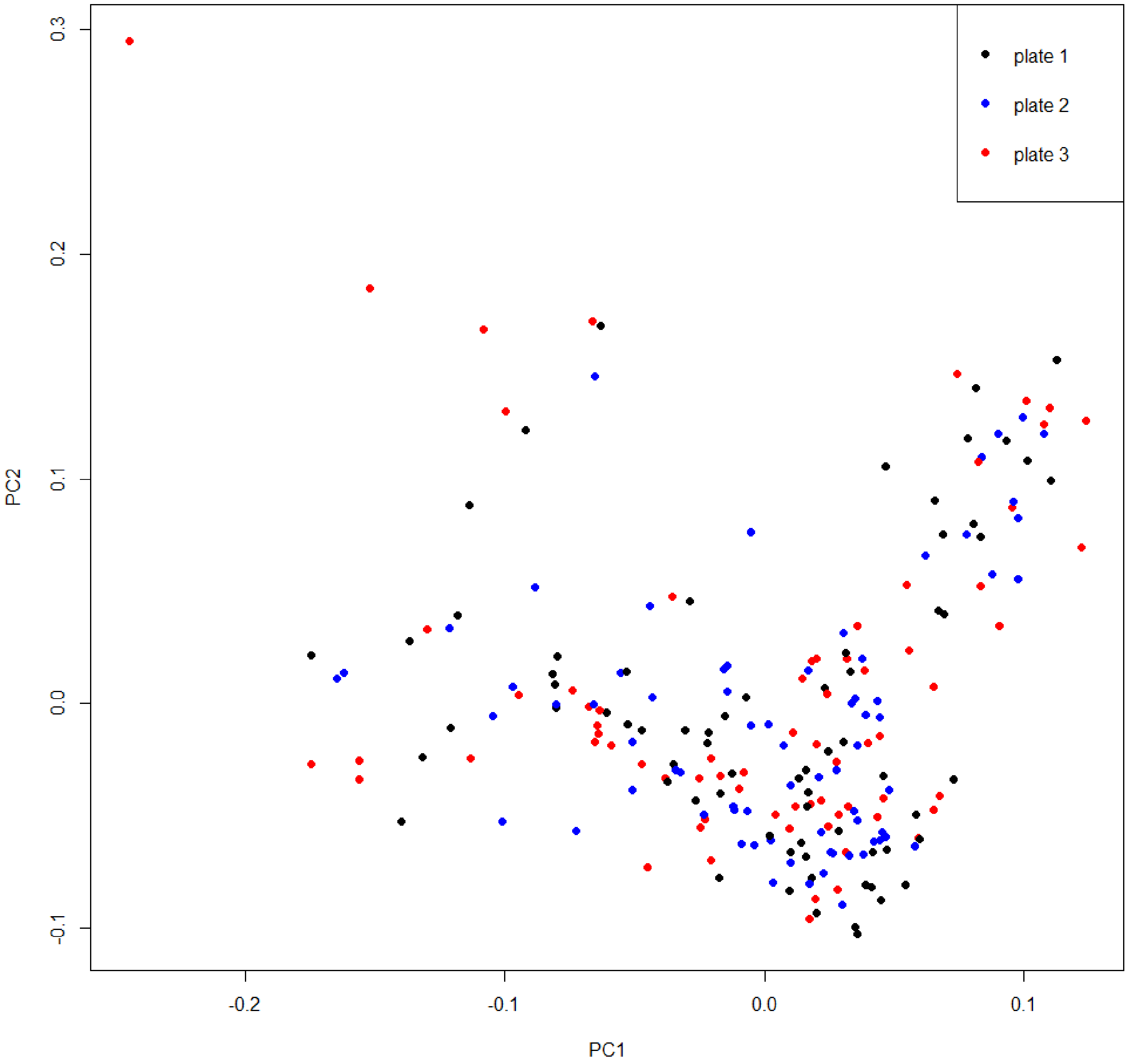

Supplement: Supplementary file 1 [file genes-13-00650-s001.zip › Figure S1.pdf]
