# Supplementary material for: Genomic and Transcriptomic Characterization of Atypical Recurrent Flank Alopecia in the Cesky Fousek
Source: Genes (Basel). 2022 Apr 7;13(4):650. doi: 10.3390/genes13040650 (PMC9033119; doi:10.3390/genes13040650)

Figure S2: Manhattan and Quantile-Quantile Plot of quantitative GWAS. The top SNP p-value does not reach the significance threshold (Bonferroni correction = $5.8 x 10^{-7}$; shown as a purple line), however, there were identified many SNPs on chr8 (colored in green) in a close proximity to each other. We consider these association suggestive. Lambda value shows that stratification correction worked well.

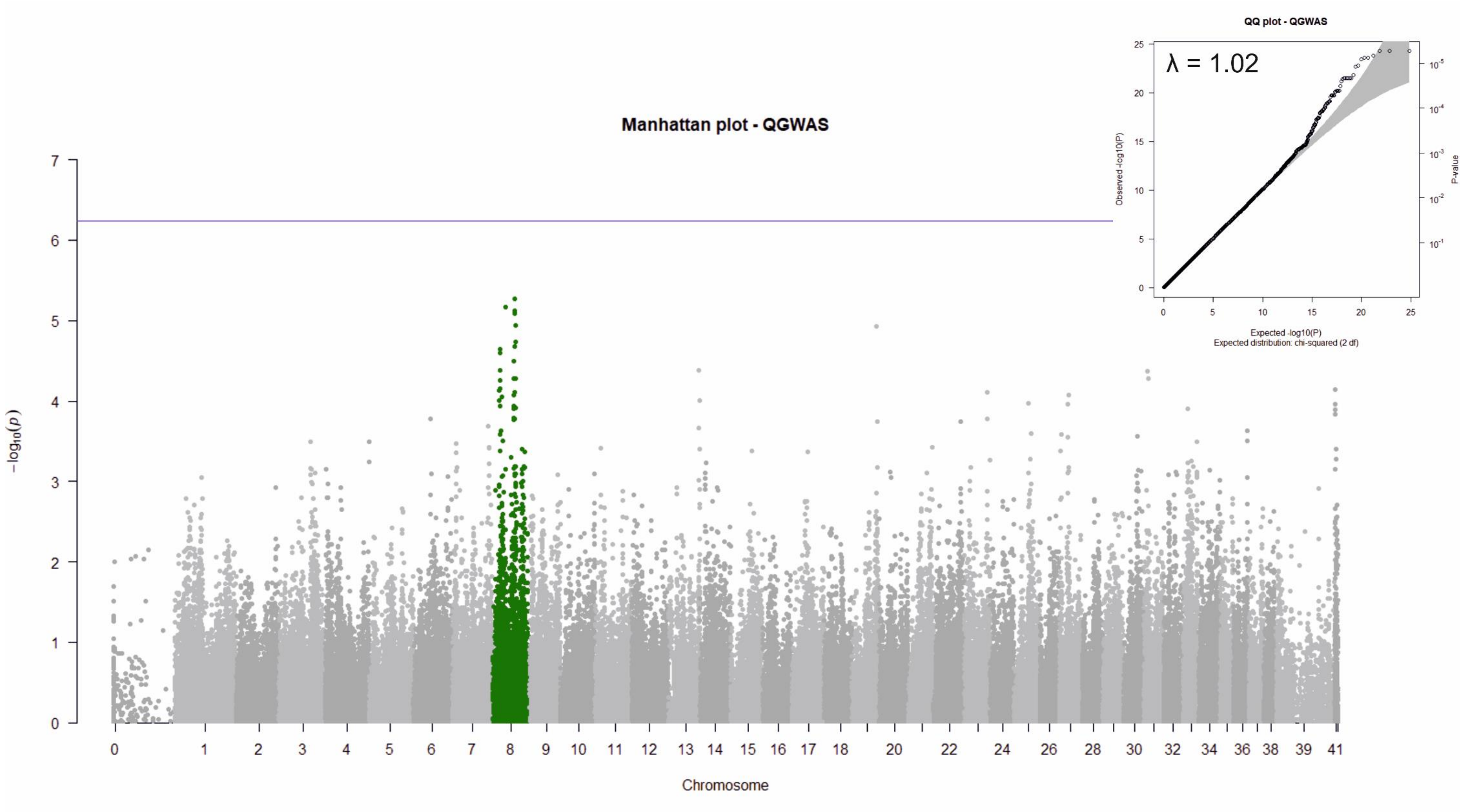

Supplement: Supplementary file 1 [file genes-13-00650-s001.zip › Figure S2.pdf]
