# Supplementary material for: Genomic and Transcriptomic Characterization of Atypical Recurrent Flank Alopecia in the Cesky Fousek
Source: Genes (Basel). 2022 Apr 7;13(4):650. doi: 10.3390/genes13040650 (PMC9033119; doi:10.3390/genes13040650)

Figure S3: Manhattan and Quantile-Quantile Plot of GWAS analysis of individuals affected up to two years of age. Identified variants could have an effect on the time and/or severity of the affection. One variant can be considered significant (chr21, BICF2G630640798, raw P-value = 5.01×10^{-7}), it reaches the significance threshold (Bonferroni correction = 5.8×10^{-7}; shown as a purple line), and it lies in a gene ANO3. Lambda value shows that stratification correction worked well.

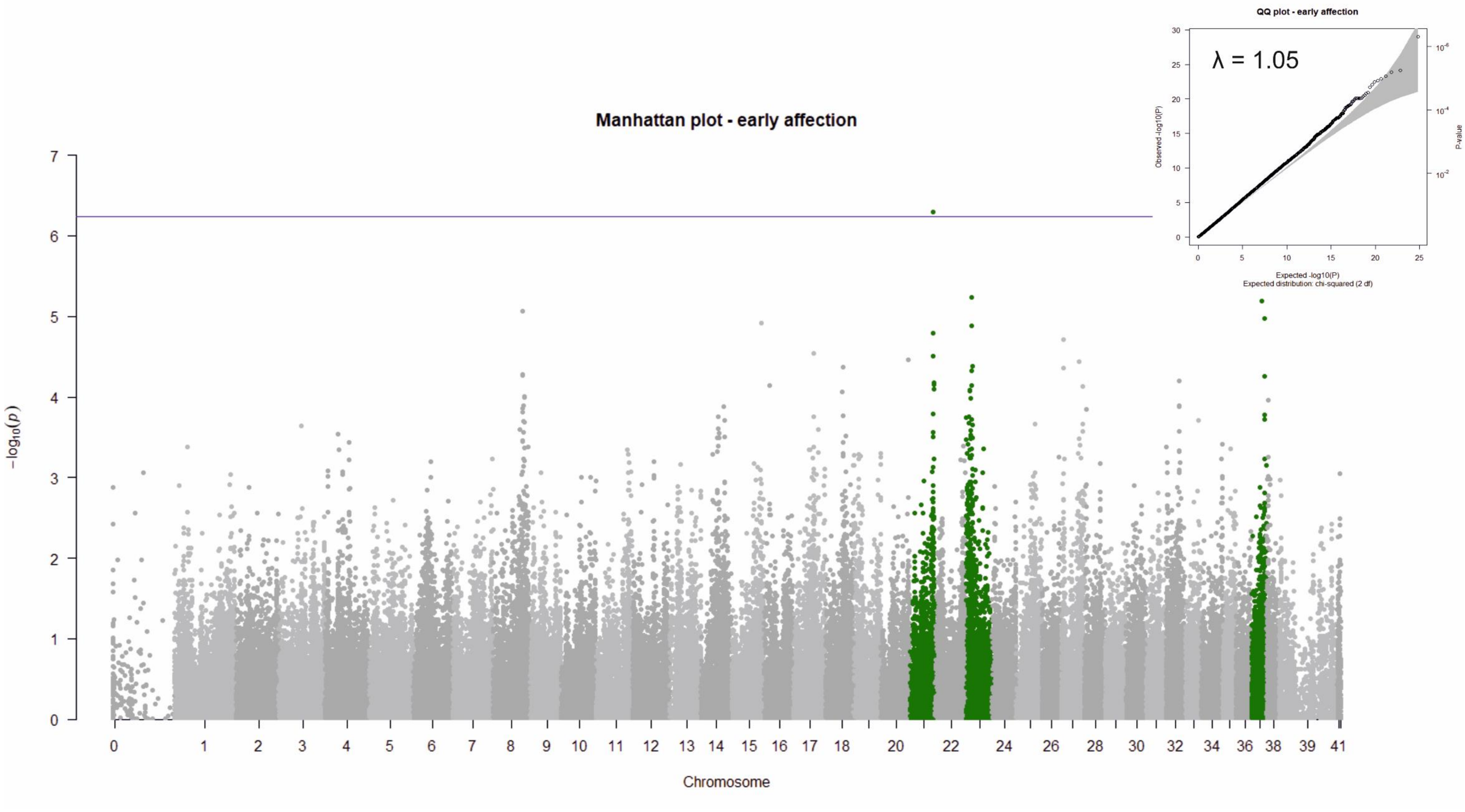

Supplement: Supplementary file 1 [file genes-13-00650-s001.zip › Figure S3.pdf]
