# Supplementary material for: Genomic and Transcriptomic Characterization of Atypical Recurrent Flank Alopecia in the Cesky Fousek
Source: Genes (Basel). 2022 Apr 7;13(4):650. doi: 10.3390/genes13040650 (PMC9033119; doi:10.3390/genes13040650)

Figure S4: Manhattan and Quantile-Quantile Plot of GWAS analysis of individuals with severe affection (level 4 aRFA). In green are colored chromosomes carrying top three SNPs. The significance threshold was based on Bonferroni correction (5.8×10^{-7}; shown in a purple line). The top SNP does not reach the significance threshold, however it is the same SNP as in the early onset GWAS. Lambda value shows that stratification correction worked well.

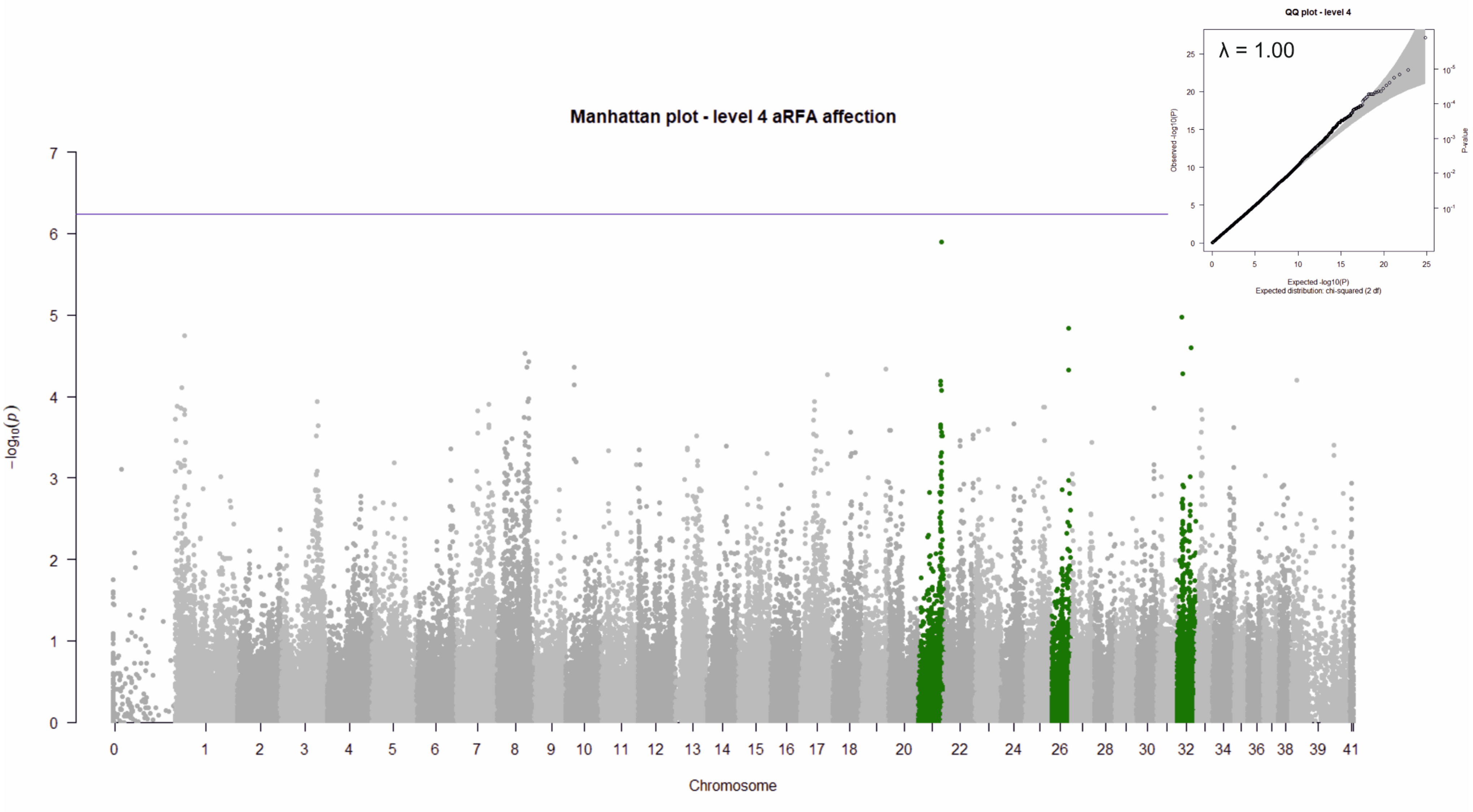

Supplement: Supplementary file 1 [file genes-13-00650-s001.zip › Figure S4.pdf]
