# Supplementary material for: Genomic and Transcriptomic Characterization of Atypical Recurrent Flank Alopecia in the Cesky Fousek
Source: Genes (Basel). 2022 Apr 7;13(4):650. doi: 10.3390/genes13040650 (PMC9033119; doi:10.3390/genes13040650)

Figure S5: Linkage disequilibrium (r2) plots for top SNP on chromosomes 19 and 8 from the case/control GWAS. The plots show LD among the top SNP on the chromosome and other SNPs within 2Mb or 4Mb (black-no LD; blue – weak LD, green – intermediate LD, yellow – moderate LD; red – strong LD).

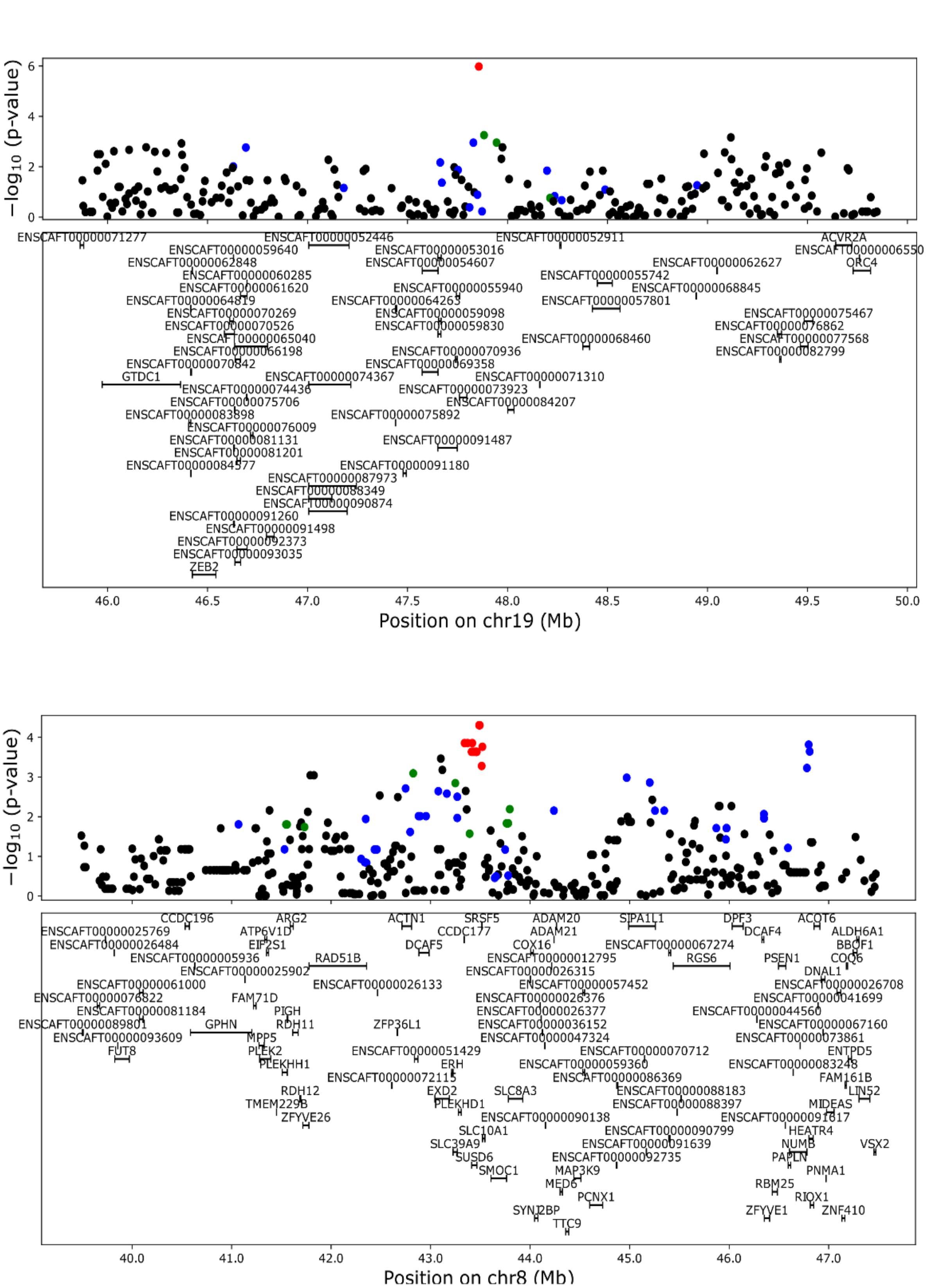

Supplement: Supplementary file 1 [file genes-13-00650-s001.zip › Figure S5.pdf]
