# Supplementary material for: Genomic and Transcriptomic Characterization of Atypical Recurrent Flank Alopecia in the Cesky Fousek
Source: Genes (Basel). 2022 Apr 7;13(4):650. doi: 10.3390/genes13040650 (PMC9033119; doi:10.3390/genes13040650)

Figure S6: Predicted protein-protein interactions among the 144 GWAS candidate genes' products. The GWAS gene candidates showed enriched protein-protein interactions, specifically, they were predicted to be connected in six clusters of more than three proteins. Each cluser is connected through the lines of different thickness, that represent the strength of evidence of functional connection between genes. Color of each gene have not been changed from the STRING database output.

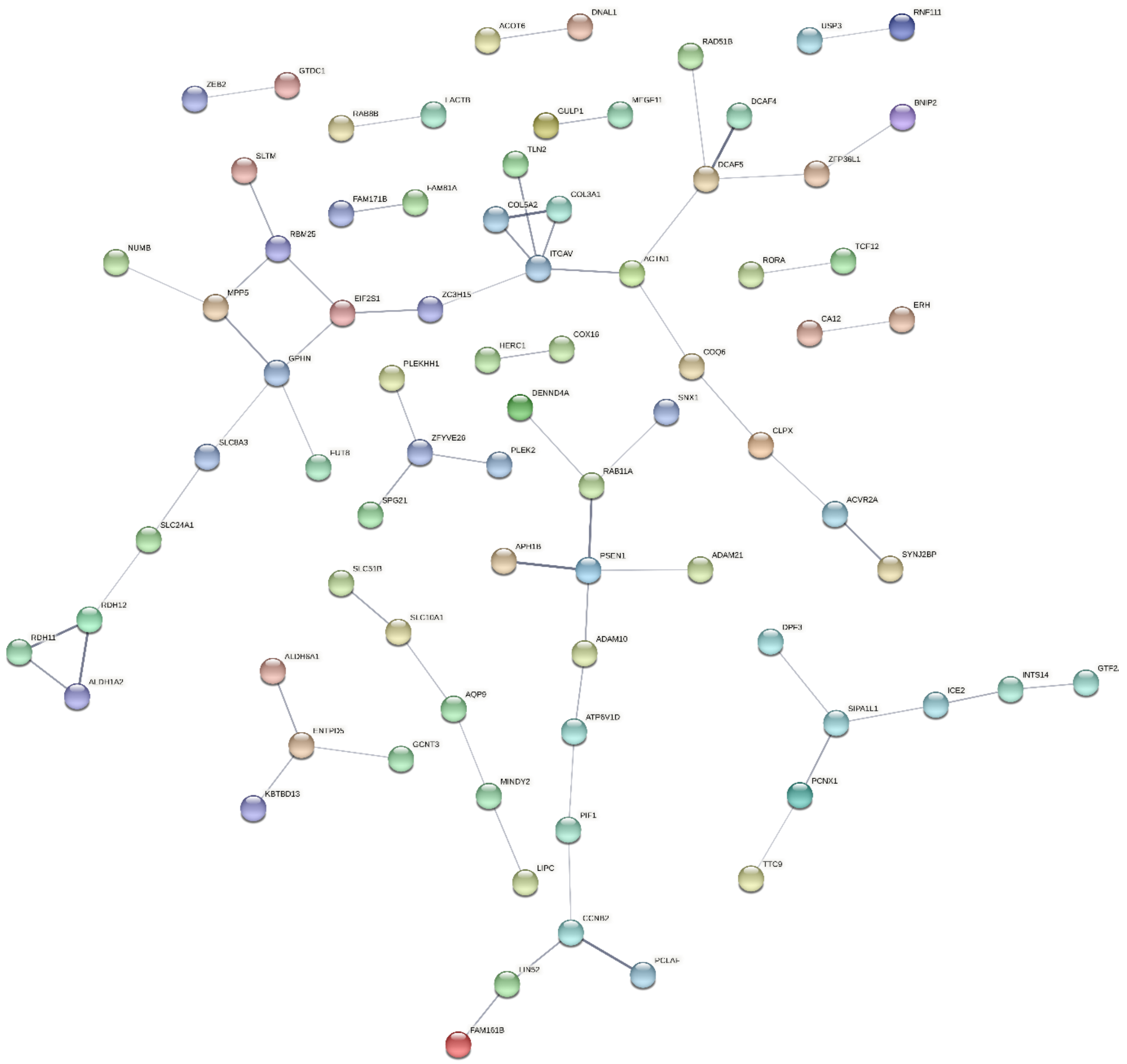

Supplement: Supplementary file 1 [file genes-13-00650-s001.zip › Figure S6.pdf]
