# Supplementary material for: Genomic and Transcriptomic Characterization of Atypical Recurrent Flank Alopecia in the Cesky Fousek
Source: Genes (Basel). 2022 Apr 7;13(4):650. doi: 10.3390/genes13040650 (PMC9033119; doi:10.3390/genes13040650)

Figure S7: Interactions of GWAS candidate genes (green) and STRING-associated strongly differentially expressed genes, colored by their level of expression. Only medium and higher confidence associations were used (increasing thickness of lines connecting genes indicates greater confidence). Colorful bubbles represent the metabolic pathways common for each cluster of genes. Shown are also couples and smaller clusters of interacting genes.

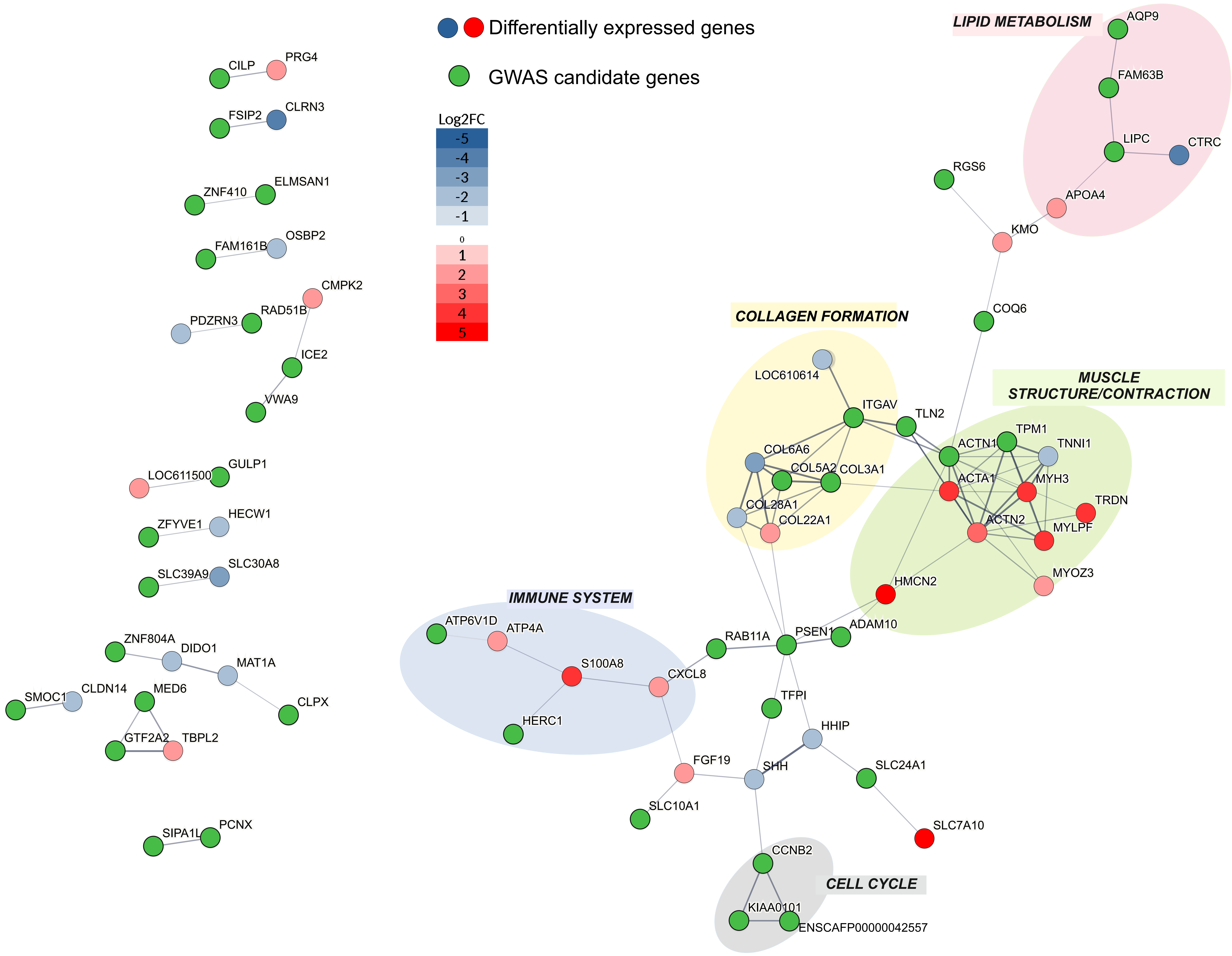

Supplement: Supplementary file 1 [file genes-13-00650-s001.zip › Figure S7.pdf]
