# Supplementary material for: Genomic and Transcriptomic Characterization of Atypical Recurrent Flank Alopecia in the Cesky Fousek
Source: Genes (Basel). 2022 Apr 7;13(4):650. doi: 10.3390/genes13040650 (PMC9033119; doi:10.3390/genes13040650)
Supplement: Supplementary file 1 [file genes-13-00650-s001.zip › Table S2.pdf]

Table S2. Summary of patient data from animals included in the study for transcriptome analysis. Animals were included or excluded from RNAsequencing analysis based on presence of consistent histological features and clinical picture.

Dog Number	Group	Age at time of biopsy	Sex
B2	Control	6 y 2 mo	F
B5	Control	10 y 5 mo	M
B6	Control	7 y 7 mo	F
B12	Control	7 y 9 mo	F
B13	Control	3 y 2 mo	F
B14	Control	1 y 4 mo	F
B7	Alopecia	9 y 2 mo	F
B9	Alopecia	10 y 11 mo	F
B10	Alopecia	3 y 11 mo	F
B11	Alopecia	1 y 11 mo	F
B3	Alopecia	9 y 4 mo	F
