# Supplementary material for: Genomic and Transcriptomic Characterization of Atypical Recurrent Flank Alopecia in the Cesky Fousek
Source: Genes (Basel). 2022 Apr 7;13(4):650. doi: 10.3390/genes13040650 (PMC9033119; doi:10.3390/genes13040650)
Supplement: Supplementary file 1 [file genes-13-00650-s001.zip › Table S3.pdf]

Table S3: Results of one of the additional GWAS analyses for two groups of individuals - healthy individuals of age 10+ and alopecic individuals with severe affection (level 4 aRFA). The top SNP on chromosome 21 is the same SNP as in analysis of early affection (Table 5), however, the significance level is not met.

Chr	SNP name	Position (bp)	Allele Freq	P-value
21	BICF2G630640798	47085771	0.204	1.28x10-06
32	BICF2P976235	8550630	0.463	1.07x10-05
26	BICF2P946509	33145128	0.093	1.46x10-05
1	BICF2G630715088	19568314	0.426	1.77x10-05
32	BICF2P907131	27577699	0.065	2.52x10-05
8	BICF2P1386913	55523835	0.370	2.97x10-05
8	BICF2S23448109	63200694	0.102	3.71x10-05
10	BICF2G630476932	18071694	0.213	4.37x10-05
8	chr8_59707832	59707832	0.204	4.40x10-05
19	BICF2G630254010	49287352	0.259	4.62x10-05
26	BICF2P1246793	32942963	0.176	4.72x10-05
32	BICF2P1149618	10657030	0.296	5.29x10-05
32	BICF2P213903	10649129	0.296	5.29x10-05
32	TIGRP2P378950_rs8534752	10680407	0.296	5.29x10-05
32	chr32_10656790	10656790	0.296	5.29x10-05
17	TIGRP2P235215_rs8492674	54162595	0.426	5.36x10-05
39	BICF2G630535234	19332927	0.157	6.38x10-05
21	BICF2P300797	45020370	0.463	6.57x10-05
21	BICF2P597850	45432234	0.194	7.15x10-05
10	BICF2S23763624	18015742	0.194	7.24x10-05
