# Supplementary material for: Genomic and Transcriptomic Characterization of Atypical Recurrent Flank Alopecia in the Cesky Fousek
Source: Genes (Basel). 2022 Apr 7;13(4):650. doi: 10.3390/genes13040650 (PMC9033119; doi:10.3390/genes13040650)
Supplement: Supplementary file 1 [file genes-13-00650-s001.zip › Table S6.pdf]

Table S6: Top ten most relevant pathways identified using Reactome pathway analysis on downregulated genes.

Pathway name	Entities				Reactions	
	found	ratio	P-value	FDR	found	ratio
Gap junction assembly	10/41	0.003	5.53x10-4	0.425	16/16	0.001
Extracellular matrix organization	39/329	0.023	5.81x10-4	0.425	125/318	0.025
Oligomerization of connexins into connexons	3/3	2.08x10-4	0.001	0.425	3/3	2.40x10-4
Transport of connexins along the secretory pathway	3/3	2.08x10-4	0.001	0.425	2/2	1.60x10-4
Gap junction trafficking and regulation	11/56	0.004	0.002	0.496	24/24	0.002
GLI proteins bind promoters of Hh responsive genes to promote transcription	4/8	5.54x10-4	0.002	0.544	4/4	3.20x10-4
Gap junction trafficking	10/52	0.004	0.003	0.653	20/20	0.002
Signaling by Hedgehog	21/168	0.012	0.006	0.72	71/82	0.007
RHO GTPases activate PAKs	6/27	0.002	0.01	0.72	14/15	0.001
Hedgehog 'off' state	16/124	0.009	0.011	0.72	26/32	0.003
