# Supplementary material for: Genomic and Transcriptomic Characterization of Atypical Recurrent Flank Alopecia in the Cesky Fousek
Source: Genes (Basel). 2022 Apr 7;13(4):650. doi: 10.3390/genes13040650 (PMC9033119; doi:10.3390/genes13040650)
Supplement: Supplementary file 1 [file genes-13-00650-s001.zip › Table S7.pdf]

Table S7: Top thirteen most relevant pathways identified using Reactome pathway analysis on upregulated genes.

Pathway name	Entities				Reactions	
	found	ratio	P-value	FDR	found	ratio
Activation of gene expression by SREBF (SREBP)	26/70	0.005	1.87x10-9	2.74x10-6	24/42	0.003
Striated Muscle Contraction	19/40	0.003	6.28x10-9	4.14x10-6	4/4	3.20x10-4
Regulation of cholesterol biosynthesis by SREBP (SREBF)	26/86	0.006	1.06x10-7	4.66x10-5	24/52	0.004
Cholesterol biosynthesis	19/72	0.005	3.30x10-5	0.011	27/32	0.003
Muscle contraction	41/256	0.018	2.48x10-4	0.066	22/41	0.003
Metabolism of steroids	47/322	0.022	6.75x10-4	0.149	87/235	0.019
Interferon alpha/beta signaling	30/184	0.013	0.001	0.227	3/20	0.002
PPARA activates gene expression	27/174	0.012	0.004	0.654	41/41	0.003
Regulation of lipid metabolism by PPARalpha	27/176	0.012	0.005	0.67	42/44	0.004
Synthesis of very long-chain fatty acyl-CoAs	10/51	0.004	0.016	0.867	8/12	9.61x10-4
Class I peroxisomal membrane protein import	5/20	0.001	0.033	0.867	6/6	4.80x10-4
Cholesterol biosynthesis via lathosterol	4/14	9.70x10-4	0.036	0.867	4/4	3.20x10-4
Cholesterol biosynthesis via desmosterol	4/14	9.70x10-4	0.036	0.867	4/4	3.20x10-4
