# Supplementary material for: Genomic and Transcriptomic Characterization of Atypical Recurrent Flank Alopecia in the Cesky Fousek
Source: Genes (Basel). 2022 Apr 7;13(4):650. doi: 10.3390/genes13040650 (PMC9033119; doi:10.3390/genes13040650)
Supplement: Supplementary file 1 [file genes-13-00650-s001.zip › Table S8.pdf]

Table S8: Fisher's exact test of eleven overlaping genes from GWAS study and RNA-seq study. The overlap was found non-significant.

V1	V2
ARG2	1.039044225
HACD3	1.017925863
PAPLN	0.780319225
TPM1	0.723505465
LACTB	0.558208956
RAD51B	0.497676468
SLC25A3	0.489588717
RDH11	0.478515289
GTDC1	0.459480995
SNX1	0.3261547
TRIP4	0.311368462
